# A large public dataset of annotated clinical MRIs and metadata of patients with acute stroke

**DOI:** 10.1038/s41597-023-02457-9

**Published:** 2023-08-22

**Authors:** Chin-Fu Liu, Richard Leigh, Brenda Johnson, Victor Urrutia, Johnny Hsu, Xin Xu, Xin Li, Susumu Mori, Argye E. Hillis, Andreia V. Faria

**Affiliations:** 1 Center for Imaging Science, Johns Hopkins University, Baltimore, MD USA; 2 Department of Biomedical Engineering, Johns Hopkins University, Baltimore, MD USA; 3 Department of Neurology, School of Medicine, Johns Hopkins University, Baltimore, MD USA; 4 Department of Radiology, School of Medicine, Johns Hopkins University, Baltimore, MD USA; 5 Department of Physical Medicine & Rehabilitation, and Department of Cognitive Science, Johns Hopkins University, Baltimore, MD USA

**Keywords:** Translational research, Databases, Stroke

## Abstract

To extract meaningful and reproducible models of brain function from stroke images, for both clinical and research proposes, is a daunting task severely hindered by the great variability of lesion frequency and patterns. Large datasets are therefore imperative, as well as fully automated image post-processing tools to analyze them. The development of such tools, particularly with artificial intelligence, is highly dependent on the availability of large datasets to model training and testing. We present a public dataset of 2,888 multimodal clinical MRIs of patients with acute and early subacute stroke, with manual lesion segmentation, and metadata. The dataset provides high quality, large scale, human-supervised knowledge to feed artificial intelligence models and enable further development of tools to automate several tasks that currently rely on human labor, such as lesion segmentation, labeling, calculation of disease-relevant scores, and lesion-based studies relating function to frequency lesion maps.

## Background & Summary

Stroke is the 5th more frequent cause of death and a leading cause of long-term disability in the United States^1^. Extracting meaningful and reproducible models of brain function from stroke images is a daunting task severely hindered by the great variability of lesion frequency and patterns. A corollary to this problem is that large datasets are imperative to encompass the possible lesion-function relationships. While biomedicine has seen a shift from “anecdotal” experiences to objective, data-supported evidence based on large amounts of data, many lesion-based studies failed to weather this transition, as evidenced by a plethora of underpowered designs leading to inexact extrapolations, or to conclusions that cannot be validated on external populations^2–6^. In addition, technical developments with artificial intelligence (AI) depend on the availability of high quality, large scale, human-supervised dataset to generate and test meaningful and reproducible models^7–9^. Although unsupervised and self-supervised techniques can extract valuable insights from unannotated data, their success still currently depends on the specific task, as well as the quality and quantity of available data. For instance, domain adaptation or transfer learning from unsupervised models, or from models trained with unrelated data (e.g., Large Language Model Meta AI models, LLAMA2^10^) might be highly efficient only if fine-tunned with expert labeled data. Therefore, combining these methods with expert-annotated data can further improve the accuracy and reliability of AI models in medical imaging applications.

A public dataset of acute stroke MRIs, associated with lesion delineation and organized non-image information will potentially enable clinical researchers to advance in clinical modeling and prediction. It will also enable the bioengineering community to develop and test AI algorithms of technical and clinical relevance, e.g., for lesion segmentation, brain mapping, and automatic generation of labels and scores. AI applications in various diseases, such as in chest X-ray, dermatology and histopathology images, and detection of breast cancer in mammography, have drastically increased, due to the availability of large image datasets^11–16^. Brain MRIs, particularly in acute conditions, offer extra challenges to the organization of large datasets, such as the lack of data (MRI scan is costly, therefore less common), the large variability among scanners and protocols, and the volumetric nature of the data which hinders annotation and expert labeling. As of today, the most successful examples of open-source collections of annotated MRIs are probably the brain tumor dataset of 750 patients included in the Medical Segmentation Decathlon (MSD)^17^, used in the Brain Tumor Image Segmentation (BraTS) challenge, and the FastMRI+^18^, a collection of about 7 thousand brain MRIs, with diverse pathologies, some of them with bound-box 2D annotation. In acute stroke, the lack of such large, annotated, high quality dataset, rather than mathematical or computational resources, is the current bottleneck for AI development.

The first efforts to create stroke repositories started with population-based epidemiological studies^19,20^ and did not include images. Starting in the 2,000’s, both the medical and the bioengineering communities acknowledged the need for a central repository for acute stroke images, in addition to metadata. Initiatives such as the “Acute Stroke Imaging Research Roadmap”^21^ initiated such effort, with the goal of standardizing imaging techniques, accessing the accuracy and clinical utility of imaging markers, and validating imaging biomarkers relevant to clinical outcomes. Since then, various consortiums and trials^22–27^ were able to accumulate large amounts of data, often available “after competition” and/or “upon request”. These conditions, however, do not guarantee that the data are shared under’FAIR’ principles^28,29^. FAIR stands for Findable, Accessible, Interoperable, and Reusable. Findable data are assigned persistent identifiers and well-described metadata, ensuring their easy discovery. Accessible data are openly available with clear access protocols, promoting transparency and inclusivity. Interoperable data are structured in a way that facilitates integration across diverse platforms and tools, enabling seamless collaboration and analysis. Lastly, reusable data are properly documented, allowing researchers to effectively understand, reproduce, and build upon previous work. Embracing the FAIR principles not only accelerates scientific discovery but also fosters a culture of responsible and efficient data sharing within the research community. A search in generalist repositories (e.g., Dataverse, Mendeley Data, Dryad, Open Science Framework, Vivli) or using tools suited to find “open data” (e.g., Google Dataset Search, Data Citation Index, Data.gov) mostly reveals end-analysis data that do not serve purposes such as technical development. In addition, datasets from published studies usually involve a modest number of subjects and are research-focused, acquired with homogeneous and particular protocols that do not reflect the noise and variability of clinical data, hindering the translational potential.

We share the first annotated large dataset of clinical acute stroke MRIs, associated to demographic and clinical metadata. Recently, a dataset of (mostly) chronic stroke lesions annotated in high resolution

T1-WIs (ATLAS^30^, followed by ATLASv2^31^) under the ENIGMA Stroke Recovery initiative^32^ was well received by the neuroscience and bioengineering communities. The ATLAS has been used to improve lesion segmentation of chronic lesions in high resolution images, to create new tools for processing chronic stroke MRIs, as well as for education proposes^33,34^. Acute stroke MRIs, however, require specialized processing because of the particular lesion intensity characteristics, the images low-resolution, heterogeneity, and noise. The organizers of the Ischemic Stroke Lesion Segmentation Challenge 2022 (ISLES22) recently released 250 MRIs with acute stroke masks^35^. An analogous large, independent, multi-modality and clinical-representative dataset of acute strokes is highly anticipated.

The resource we present consists of 2,888 clinical MRIs of patients admitted with acute or early subacute stroke. It includes diverse protocols and MRI modalities, with typical clinical resolution. The large sample, as well as the technical and population heterogeneity, improve the potential generalization of models developed with these data. It includes diverse metadata, comprised of demographic information, basic clinical profile (including National Institutes of Health Stroke Scale (NIHSS) scores, 90 days follow up modified Rankin Score (mRS), hospitalization duration, biometric screening at hospital admission and discharge, and associated health conditions), and expert description of the acute lesion. The stroke lesion is manually defined in the diffusion weighted images (DWI); the images are provided in native subject space and in standard space (Montreal Neurological Institute, MNI). The data format and organization follows the Brain Imaging Data Structure, BIDS^36^ guidelines, facilitating navigation and sharing. To the best of our knowledge, this is the first large clinical MRI dataset shared under FAIR principles, and is available at the Inter-university Consortium for Political and Social Research, ICPSR (https://www.icpsr.umich.edu/web/ICPSR/studies/38464)^37^.

## Methods

### Cohort

Clinical data and MRIs were obtained retrospectively from patients admitted from 2009–2019 to the Johns Hopkins Comprehensive Stroke Center. The dataset creation, under waiver of informed consent, and its sharing model followed the recommendations the Johns Hopkins Internal Review Board and were approved by this board (IRB00228775). About 500 stroke patients are admitted annually, and an estimated 70% of them have MRI at admission, the majority between 6–24 hours after symptoms. To create the dataset presented here, we included patients admitted with the clinical diagnosis of acute stroke that had MRIs with DWI. A neuroradiologist (AVF) excluded those whose scans had artifacts considered impeditive of the visual analysis, as well as post-operative or strokes secondary to etiologies other than vascular, e.g., secondary to brain tumors (15% of cases). The final dataset includes 2,888 patients.

An expert neuroradiologist (AVF) and a stroke neurologist (RL), both with more than 20 years of experience, reviewed the lesions to provide qualitative descriptions of the type of lesion and location. According to their radiological appearance at MRI, the lesions were categorized as: (1) ischemic, which are lesions primarily hyperintense in DWI and hypo/isointense in the apparent diffusion coefficient (ADC); (2) hemorrhage, when any signal of bleeding, intra or extra-parenchymal was detected, or (3) “not visible” when the stroke lesion was not visually detected. Note that hemorrhage includes hemorrhagic transformation of ischemic strokes, as well as primary intraparenchymal, subarachnoid, subdural and intraventricular hemorrhages. The category “not visible” includes mostly transient ischemic attacks (TIA) or strokes with volume bellow the image resolution. Note that the radiological classification of “lesion type” aims to facilitate image-based organization and search, and does not necessarily corresponds to the clinical diagnosis of “stroke type” (Ischemic Stroke, Embolic Stroke, TIA, Intracranial Hemorrhage, Subarachnoid Hemorrhage). The “stroke type” was recorded at patient’s admission and is also provided with the dataset.

The demographic and clinical information recorded at admission and discharge is provided for each patient, following the BIDS^36^ recommendation. The itemized description of the information available is in the “Dictionary” (Supplementary Material). The population, image and lesions profiles are listed in Table 1. The mean age of the patients was 62.16 years-old (±14.68); ages ranged from 18 to 99 years-old. There was a slightly predominance of male (52.87%) over female. African American/Black was the predominant racial group (43.52%), followed by Caucasians (31.79%). The mean NIHSS score at admission was 5.80 ± 6.48. The “hemorrhage” group had the highest mean scores, followed by the “ischemic”; the “not-visible” had the lowest. The length of hospitalization, which indirectly reflects the severity of the stroke, was 6.63 days in average. The “hemorrhage” group had the longest length, followed by the “ischemic”; the “not-visible” had the shortest. The results of the following laboratorial tests were recorded at admission: cholesterol profile, hemoglobin a1c, serum creatinine, prothrombin international normalized ratio, fasting glucose; the means are listed in Table 1. We also report blood pressure at admission, ambulation status (prior, at admission and at discharge), body mass index (BMI), and modified Rankin scores (mRS) collected by phone interview 90 days after the stroke event. The previous medical condition most often reported was hypertension (60.80%), followed by dyslipidemia (31.68%) and diabetes (25.66%). We report time from symptoms to scan in patients who were (or whose caregiver was) highly confident about symptoms onset. In most of cases, the MRI was performed 6 or more hours after the initial symptoms. The MRI scan was performed after acute treatment (intravenous tissue plasminogen activator, ivtPA, in the majority, followed by thrombolysis) in 43.32% of patients.

**Table 1:** Demographic and clinical profile of the population, MRI and lesion characteristics.

Dataset		Total	Ischemic	Hemorrhagic	Not Visible
		(n = 2888)	(n = 1878, 70%)	(n = 540, 12%)	(n = 470, 18%)
**Demographics**	**Age in years**	62.00[53,73]; 0	62.00[53,72]; 0	64.00[54,75]; 0	61.00[52,71]; 0
	**Sex**				
	**Female**	1361(47.13%)	866(46.11%)	259(47.96%)	236(50.21%)
	**Male**	1527(52.87%)	1012(53.89%)	281(52.04%)	234(49.79%)
	**Race**				
	**African American**	1257 (43.52%)	824 (43.88%)	210 (38.89%)	223 (47.45%)
	**Caucasian**	918 (31.79%)	533 (28.38%)	206 (38.15%)	179 (38.09%)
	**Asian**	76 (2.63%)	44 (2.34%)	25 (4.63%)	7 (1.49%)
	**Not Recorded**	637 (22.06%)	477 (25.40%)	99 (18.33%)	61 (12.98%)
**Clinics and laboratorial tests**	**NIHSS**	3.00[1.00,8.00]; 1403	4.00[1.00,8.00]; 804	8.00[3.00,13.75]; 354	1.00[0.00,3.00]; 245
	**Systolic**	154.00[136.00,178.00]; 508	155.00[137.00,180.00]; 401	154.00[133.00,172.00]; 58	150.00[133.00,173.00]; 49
	**Diastolic**	83.00[73.00,95.00]; 508	84.00[74.00,96.00]; 401	81.00[72.00,96.75]; 58	81.00[72.00,92.00]; 49
	**Cholesterol**	167.00[137.00,201.00]; 707	168.00[137.00,203.00]; 452	162.00[134.75,194.25]; 168	168.00[140.50,202.00]; 87
	**Triglycerides**	100.00[72.00,144.50]; 717	102.00[73.00,146.00]; 460	91.00[67.75,128.00]; 168	101.00[72.00,156.00]; 89
	**HDL**	46.00[36.00,57.00]; 710	45.00[36.00,57.00]; 455	47.00[37.00,59.00]; 168	47.00[38.00,59.00]; 87
	**LDL**	95.00[70.00,124.00]; 712	96.00[71.00,125.00]; 455	92.00[67.00,115.00]; 171	95.00[69.00,124.00]; 86
	**Hemoglobin A1C**	5.80[5.40,6.60]; 805	5.90[5.40,6.70]; 514	5.70[5.40,6.50]; 178	5.80[5.40,6.40]; 113
	**Glucose**	0.90[0.80,1.20]; 509	113.00[98.00,146.00]; 451	125.00[104.00,159.00]; 79	107.00[94.00,131.00]; 59
	**Creatinine**	0.90[0.80,1.20]; 509	1.00[0.80,1.20]; 400	0.90[0.70,1.20]; 59	0.90[0.80,1.10]; 50
	**Prothrombin**	1.00[1.00,1.10]; 675	1.00[1.00,1.10]; 518	1.10[1.00,1.10]; 72	1.00[1.00,1.10]; 85
	**BMI**	27.68[24.03,32.45]; 808	27.82[24.07,32.46]; 557	27.37[23.95,31.77]; 134	27.45[24.04,33.31]; 117
	**Days at hospital**	4.00[2.00,8.00]; 829	4.00[2.00,7.00]; 567	7.00[4.00,14.00]; 123	2.00[1.00,4.00]; 139
	**Prior medical conditions**				
	**Hypertension**	1756 (60.80%)	1124 (59.85%)	346 (64.07%)	286 (60.85%)
	**Dyslipidemia**	915 (31.68%)	592 (31.52%)	147 (27.22%)	176 (37.45%)
	**Diabetes mellitus**	741 (25.66%)	481 (25.61%)	139 (25.74%)	121 (25.74%)
	**Previous stroke**	634 (21.95%)	409 (21.78%)	122 (22.59%)	103 (21.91%)
	**Smoker**	620 (21.47%)	434 (23.11%)	88 (16.30%)	98 (20.85%)
	**Atrial Fibrillation/Flutter**	272 (9.42%)	167 (8.89%)	68 (12.59%)	37 (7.87%)
	**coronary disease/prior heart infarct**	383 (13.26%)	232 (12.35%)	78 (14.44%)	73 (15.53%)
	**Obesity/Overweight**	225 (7.79%)	149 (7.93%)	35 (6.48%)	41 (8.72%)
	**Chronic renal insufficiency**	114 (3.95%)	82 (4.37%)	23 (4.26%)	9 (1.91%)
	**Family history of stroke**	111 (3.84%)	75 (3.99%)	22 (4.07%)	14 (2.98%)
	**Heat failure**	109 (3.77%)	75 (3.99%)	21 (3.89%)	13 (2.77%)
	**Migraine**	83 (2.87%)	52 (2.77%)	16 (2.96%)	15 (3.19%)
	**Sleep apnea**	57 (1.97%)	40 (2.13%)	9 (1.67%)	8 (1.70%)
	**Peripheral vascular disease**	40 (1.39%)	25 (1.33%)	6 (1.11%)	9 (1.91%)
	**Carotid Stenosis**	26 (0.90%)	17 (0.91%)	6 (1.11%)	3 (0.64%)
	**Prosthetic heart valve**	14 (0.48%)	6 (0.32%)	4 (0.74%)	4 (0.85%)
	**Sickle cell**	10 (0.35%)	6 (0.32%)	3 (0.56%)	1 (0.21%)
	**Current pregancy**	3 (0.10%)	2 (0.11%)	0 (0.00%)	1 (0.21%)
	**Hormone replacement**	3 (0.10%)	2 (0.11%)	0 (0.00%)	1 (0.21%)
	**Vein thrombosis/lung embolism**	2 (0.07%)	1 (0.05%)	1 (0.19%)	0 (0.00%)
	**Not Recorded**	665 (23.03%)	482 (25.67%)	95 (17.59%)	88 (18.72%)
	**Prior Medication**				
	**Anticholesterol**	966 (33.45%)	624 (33.23%)	174 (32.22%)	168 (35.74%)
	**Anticoagulants**	197 (6.82%)	100 (5.32%)	59 (10.93%)	38 (8.09%)
	**Antiglucose**	546 (18.91%)	361 (19.22%)	87 (16.11%)	98 (20.85%)
	**Antihypertensive**	1540 (53.32%)	974 (51.86%)	294 (54.44%)	272 (57.87%)
	**Antiplatelet**	1000 (34.63%)	630 (33.55%)	187 (34.63%)	183 (38.94%)
	**Not Recorded**	1088 (37.67%)	748 (39.83%)	195 (36.11%)	145 (30.85%)
	**90 days mRS**	2[1,3]; 1632	2[1,3]; 1018	3[1,4]; 265	1[0,2]; 348
**MRI and lesion characteristics**	**Hours from symptoms to MRI**				
	**<2/2-6/6-12/12-24 />24; missing**	81/205/234/456/398;1514	40/125/136/175/284;1118	16/24/42/111/72;275	25/56/56/170/42;121
	**MRI Magnetic Field**				
	**1.5 T**	1766 (61.15%)	1217(64.80%)	252(46.67%)	297(63.19%)
	**3.0 T**	1122 (38.85%)	661 (35.20%)	288 (53.33%)	173 (36.81%)
	**Scan manufacturer**				
	**Siemens**	2614 (90.51%)	1667 (88.76%)	517 (95.74%)	430 (91.49%)
	**Philips**	22 (0.76%)	15 (0.80%)	2 (0.37%)	5 (1.06%)
	**GE**	210 (7.27%)	166 (8.84%)	17 (3.15%)	27 (5.74%)
	**Not Recorded**	42 (1.45%)	30 (1.60%)	4 (0.74%)	8 (1.70%)
	**Treatment pre-scan**				
	**Yes; % IVtPA**	1251 (43.32%); 94%	931 (49.57%); 93.3%	98 (18.15%); 95%	222(47.23%); 95%
	**No**	1637 (56.68%)	947 (50.43%)	442 (81.85%)	248 (52.77%)
	**DWI voxel size**	5.74[3.20,7.20]	5.72[3.20,7.60]	5.74[3.20,7.20]	5.74[3.53,7.60]
	**Lesion volume in ml**	7.86[1.46,32.34]	4.27[0.98,22.12]	30.27[12.51,68.55]	N.A.
	**Hemisphere**				
	**Left**	1082 (37.47%)	834 (44.41%)	248 (45.93%)	N.A.
	**Right**	976 (33.80%)	766 (40.79%)	210 (38.89%)	
	**Bilateral**	360 (12.47%)	278 (14.80%)	82 (15.19%)	
	**Diagnosis**				
	**Embolic Stroke**	22 (0.76%)	19 (1.01%)	2 (0.37%)	1 (0.21%)
	**Intra Cerebral Hemorrhage**	381 (13.19%)	18 (0.96%)	346 (64.07%)	17 (3.62%)
	**Ischemic Stroke**	2164 (74.93%)	1794 (95.53%)	166 (30.74%)	204 (43.4%)
	**Subarachnoid Hemorrhage**	81 (2.8%)	25 (1.33%)	22 (4.07%)	34 (7.23%)
	**Transitory Ischemic Acident**	240 (8.31%)	22 (1.17%)	4 (0.74%)	214 (45.53%)

Continuous data is presented as median [interquartile range]; missing value. Categorical variables are presented by the numbers and % they represent in each group.

### MRIs

**Fig. 1:**
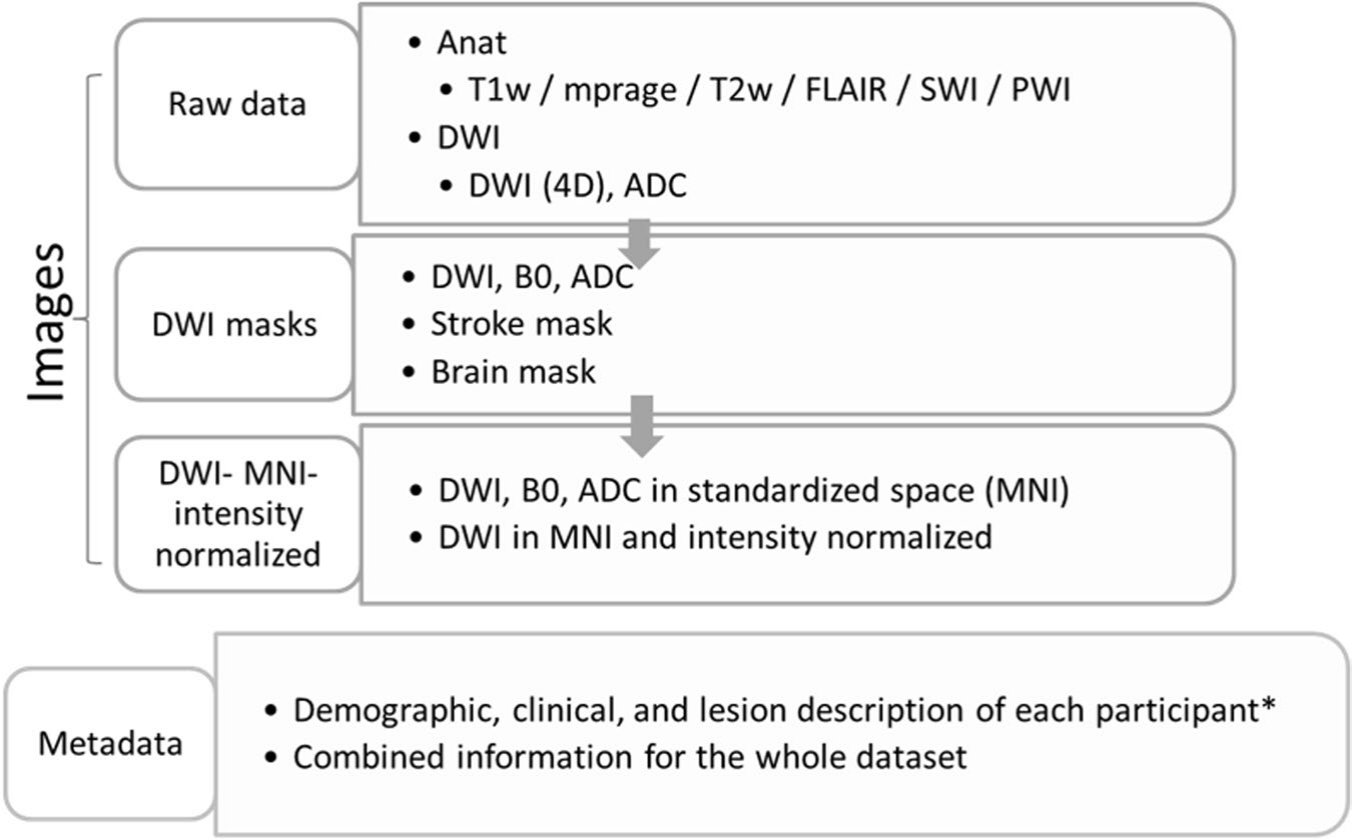
Overall description of the archive. The archive follows BIDS recommendations for structure and naming. All images are anonymized, in Nifti format. The itemized description of the metadata (* in “.tsv” format) is in the data dictionary, included in the dataset and here, as Supplementary Material. The summary of the demographic and clinical information for the cohort is in Table 1.

The MRIs were collected in 11 MRI scanners, over 10 years. This resulted in a large data variability, due to the various image protocols used over the years in different machines, scanners changes and updates, as well as modifications in acute stroke guidelines over this period. Shortly, the MRIs were performed in 1.5 T (61%) and 3 T (39%), whole-body Siemens (91.66%), Toshiba (0.21%), Phillips (7.20%), and GE (0.93%) scanners. Summaries of the acquisition parameters for all the MRI modalities in the Supplementary Table 1 and Fig. 1. The DWIs had high in plane (axial) resolution (1x1mm, or less), and typical clinical high slice thickness (2–7 mm).

Almost all patients (98.8%) had at least one MRI modality other than DWI that met the visual quality control standards and is provided with the dataset. Additional MRI sequences, and the percentage of scans that had these sequences, are: T1-weighted images (T1-WI, before or after exogenous contrast injection, n = 2,373, 82%), high resolution T1-WI MPRAGE (n = 1,298, 45%), T2-WI (n = 2,581, 90%), FLAIR (n = 2,746, 95%), Susceptibility-WI (SWI, n = 2,106, 73%), and Perfusion-WI (PWI, originally 34.2%; but only n = 531, 18.4% had “readable” quality PWIs, over passing the quality-control check).

The images were fully de-identified by removing all HIPAA (Health Insurance Portability and Accountability)-protected health information direct and indirect identifiers. The original DICOM files were converted to Neuroimaging Informatics Technology Initiative, Nifti format (nii.gz/json) using dcm2niix (https://github.com/rordenlab/dcm2niix) with the anonymization option according BIDS guide-lines. Note that the “.json” preserves the technical information from the image header. All the high resolution T1-WI MPRAGE, and the low resolution T1-WIs and FLAIR with full head coverage were defaced using FSL (https://surfer.nmr.mgh.harvard.edu/fswiki/mrideface). Another round of visual quality control was preformed to secure complete anonymization, including 3D reconstruction of each image to guarantee impossibility of face recognition. The overall structure of the archive is represented in Fig. 1 and detailed in the sections below, as well as in the “Data Availability”.

### Lesion masks

Although there is no perfect method for defining the lesion core, we chose to use DWI and Apparent Diffusion Coefficient maps (ADC), based on the fact that DWI is the most informative and most common sequence performed for acute stroke detection. Likewise, prior acute stroke studies and trials defined the lesion core in DWI, so our data will be broadly comparable to those investigations. As the majority of MRIs are performed 6 or more hours after symptoms, the odds of significant change in the lesion volume is low^38^. Nevertheless, we tabulated the time between symptoms onset and the MRI and make it available, so one can estimate the stability of the DWI-defined lesion.

**Fig. 2:**
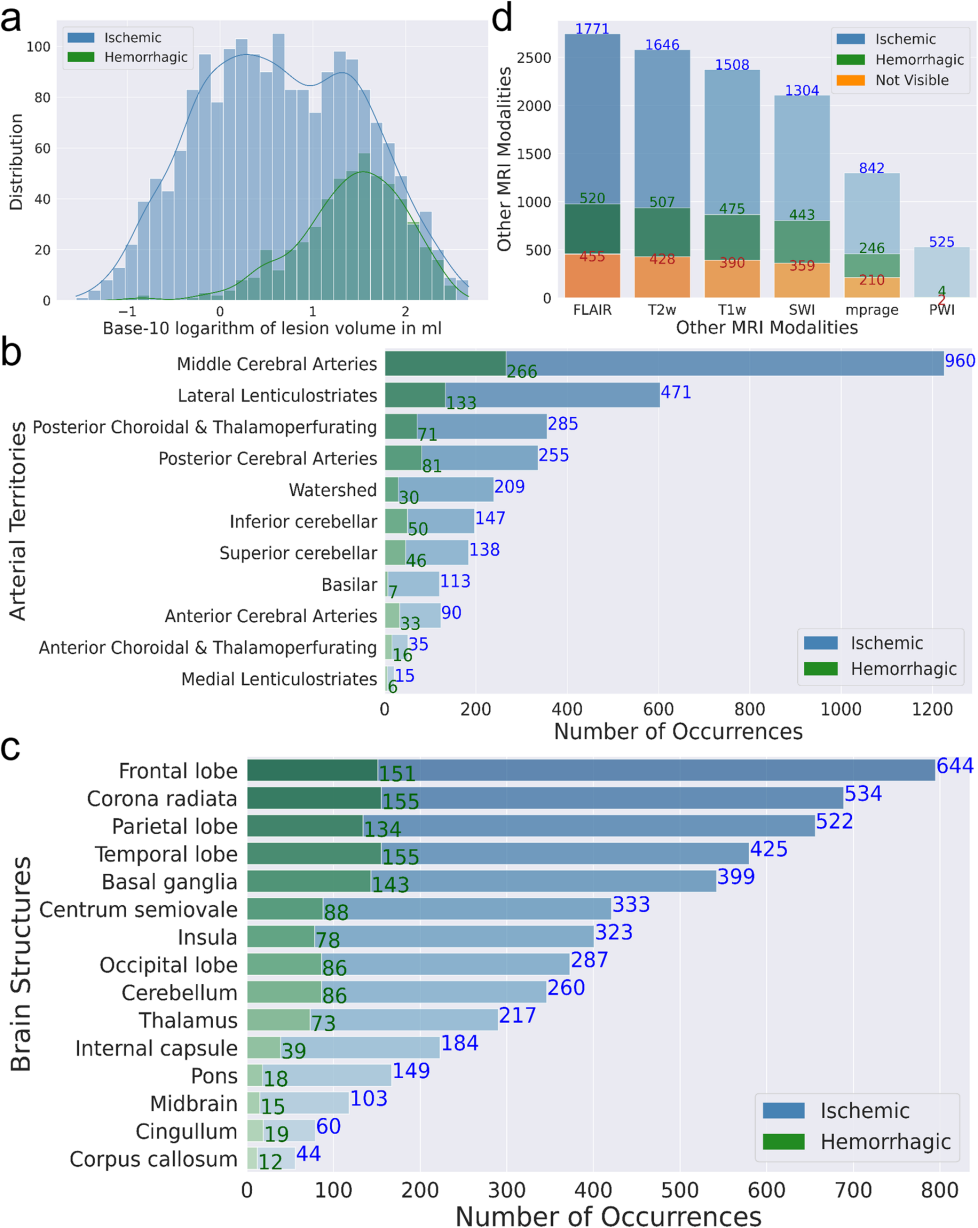
Dataset lesion and image profiles. Distribution of lesions attributed to ischemia or hemorrhage according to (**a**) volumes, (**b**) arterial territories, (**c**) brain structures. (**d**) Presence of MRI modalities other than DWI. Note that although the categorization in arterial territories is not necessarily meaningful for hemorrhage, we show it for the sake of a uniform description.

The methodological description of the lesion delineation procedures, inter- and intra-rater reliability measures, and additional technical validation of the lesion tracings are reported in “Technical Validation”. We note that the lack of ground truth for segmentation is a well know problem in imaging analysis and that lesion tracing is a subjective process, even across trained evaluators. This reinforces the importance of our exhaustive revision and final definition by consensus. Figure 2 shows the distribution of lesions according volumes and location. As shown in Table 1, the intra-parenchymal hemorrhagic lesions were significantly larger than the ischemic lesions. There was a slight predominance (not significant) of lesions in the left hemisphere compared to the right. The volumes of ischemic lesions showed a significant correlation with NIHSS (r = 0.57; p-value < 0.0001), as expected.

### “Post-processed” images

Image mapping to common coordinates (e.g., to standard templates) and intensity normalization are common steps required in most pipelines for imaging processing. To expand the access to the dataset, in addition to the native data, we offer the DWI (plus B0 and ADC) and the stroke and brain masks mapped to standard MNI space. In order to convert the images to standard coordinates, we: (1) Resampled DWI, B0, and ADC into 1 × 1 × 1 *mm*^3^; (2) Skull-stripped with an in-house “UNet BrainMask Network”^39^; (3) Used sequential linear transformations^40^ to map B0 (less affected by the acute stroke) into JHUMNIB0^41^, a template in MNI space.

Using the resulting transformation matrix, the brain and the lesion mask were registered to the MNI template by nearest neighborhood interpolation, to keep their binary nature. Because these are clinical low resolution images, with high slice thickness and, often, a fair amount of tilt on the z-axis (as they are axial oriented), the regular steps for linear transformation tend to perform less well than they do on high resolution images. Therefore, two rigorous steps of quality control were performed on the MNI-converted images: one qualitative, by visual analysis, and the other quantitative, based on how the global brain contour fits the template, as detailed in the Technical Validation section.

Regarding to the intensity normalization, it is unlikely that we can offer all the possible options that are ideal for each specific study. Some of these options are straightforward and can be easily generated by users (e.g., normalization using z-scores or maximum intensity). Others (e.g., by self-supervised methods), can be prospectively derived for particular studies using this resource. We opted by offering DWIs normalized by a method that proved successful in homogenizing images across different lesion types, in minimizing major differences in scanners (e.g. magnetic field), and in reducing the complexity and time to train Deep Learning Networks for lesion segmentation (Table 2), as detailed in in the Technical Validation section.

**Table 2:** Accuracy of the same UNET Deep Learning model on segmenting ischemic core, using DWIs normalized by different methods.

	ProposedNorm	StandardNorm	BrainMaskStandardNorm	MaxMinNorm
Validation	0.75(0.17); 0.80	0.64(0.22); 0.70	0.48(0.29); 0.51	0.54(0.24); 0.61
Testing	0.74(0.20); 0.80	0.64(0.21); 0.70	0.53(0.31); 0.62	0.53(0.23); 0.60

The normalization methods tested were: ‘ProposedNorm’: described in this manuscript; ‘StandardNorm’: standard z-score normalization on whole images; ‘BrainMaskStandardNorm’: standard z-score normalization on brain-masked region only; ‘MaxMinNorm’: Max-Min normalization. Except for the intensity normalization, all procedures for training the network, inferencing predicts, and the post-processing are the same (as in^39^). The numbers represent the average (standard deviation); media of Dice scores between the automatically and manually traced images, in the 5-fold cross-validation (total training sample = 1849) and testing samples (499).

### Probabilistic maps of lesions and “radiological normal” templates

**Fig. 3:**
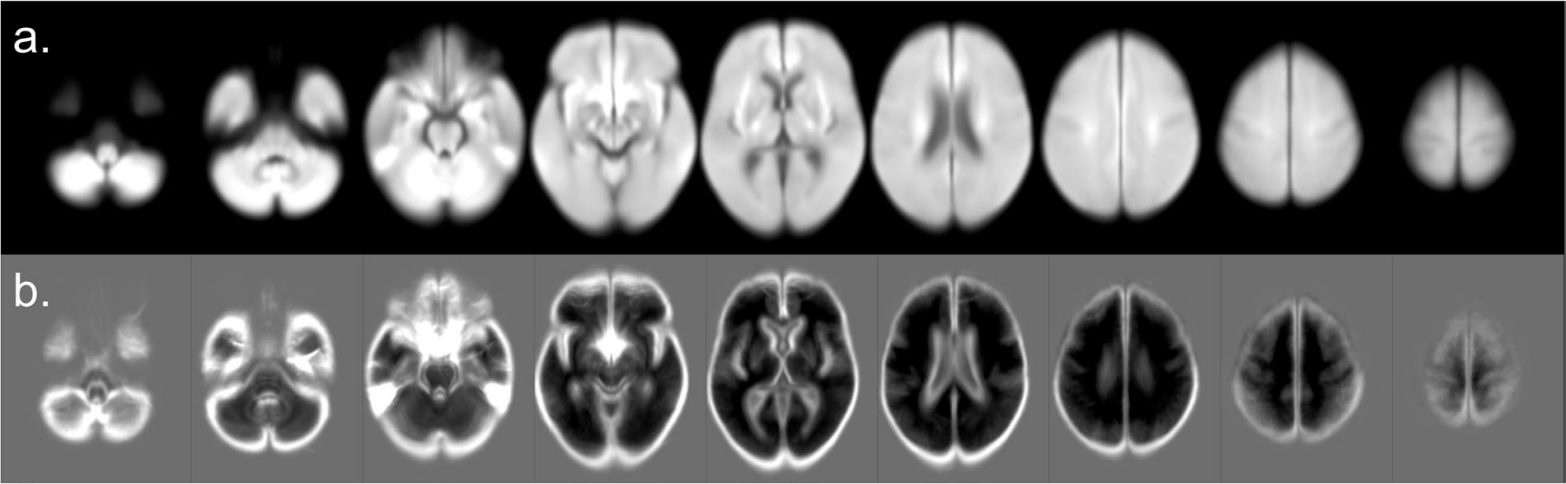
Radiological normal DWI templates. (**a**) average and (**b**) standard deviation maps of the intensity-normalized DWIs in standard space (MNI) of the cases classified as “not-visible” strokes.

Using the intensity-normalized DWIs in standard space (MNI) of the cases classified as “not-visible” strokes, we created average and standard deviation maps, here called “radiological normal” templates (Fig. 3). We note that “radiological normal” is an imperfect name, as these cases may still have abnormalities not directly related to the current stroke episode, such as white matter microvascular chronic lesions or brain atrophy. Nevertheless, such templates are representative of the radiological aspect of the brain tissue not directly affected by the acute stroke, of our population. Such templates are potentially useful for technical development, e.g., for modeling voxel classification by intensity.

**Fig. 4:**
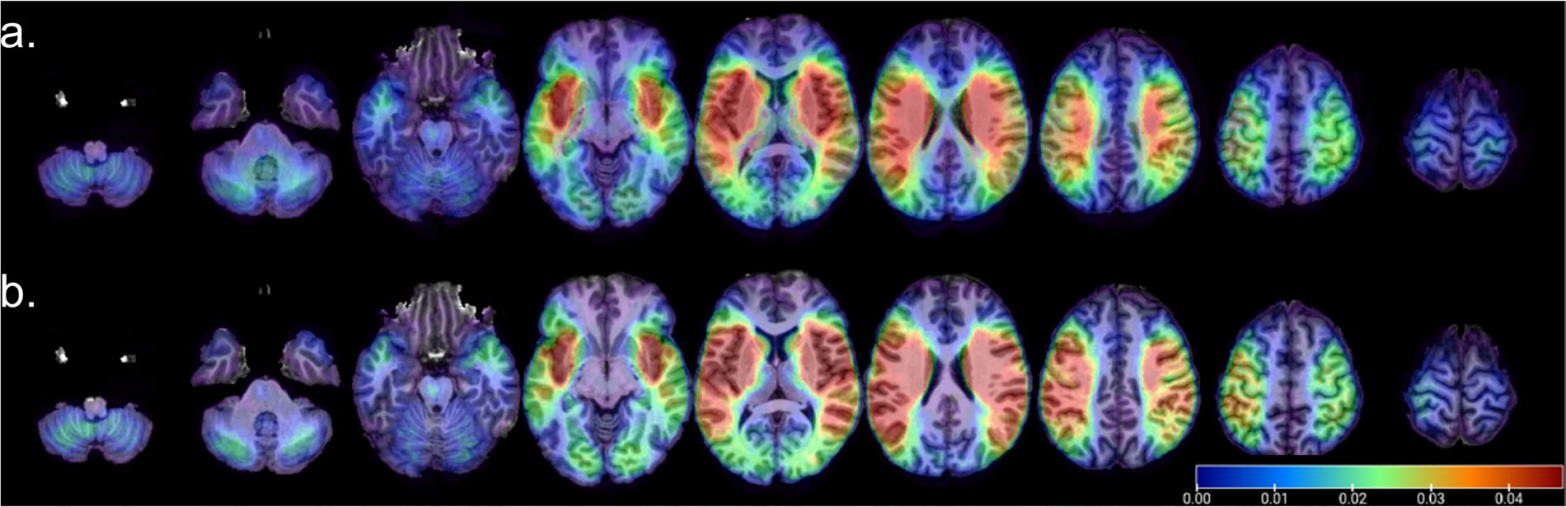
Distribution of (**a**) hemorrhage (intra-parenchymal) and (**b**) ischemic lesions in the dataset.

We also created frequency maps of the “ischemic” and inrta-parenghymal “hemorrhage” lesions, with the simple purpose of visualizing the distribution of lesions across the dataset (Fig. 4). We performed a population-based averaging of the lesion masks in MNI space, producing a voxel-wise map where values can range from 0 at each voxel (no lesion in any subject) to 1 (100% presence of the lesion across subjects). The frequency maps and the “radiological normal” templates are provided with the dataset. Because the individual lesion masks are available, users are able to create multiple other types of templates and atlases that best fit their interest.

## Data Records

The dataset is deposited in ICPSR^37^ (10.3886/ICPSR38464). Because these data were originally assembled under a waiver of patient consent, the dataset is released as a restricted-use collection under a Data Use Agreement (DUA). Each subject is identified by an 8-digit random code. The data structure, format, and naming follow the BIDS guidelines, and is as follows (see Fig. 1):

1. The main folder “raw-data” contains the image data (nii.gz and json files) in the native space of each subject in two subfolders:
  “DWI”, with 4D DWI/B0 and ADC
  “anat”, with T1-WI, MPRAGE, T2-WI, FLAIR, PWI, SWI
2. The folder “DWI-mask” contains images in native space of manually-defined lesion masks
  brain masks
  3D DWI, B0, and “recalculated” ADC
3. The folder “DWI-MNI-IntensityNormalized” contains images in standard MNI space, mapped to JHU_SS_MNI template^41^, of
  DWI, B0, ADC, lesion mask, brain mask Intensity-normalized DWI
4. The folder “phenotype” contains
  Individual files with structured metadata of each subject
5. The folder “templates” contains the following images in MNI space, according to JHU_SS_MNI template^41^
  Average and standard deviation of “radiological normal” DWIs.
  Frequency maps of ischemic and hemorrhage lesions.
6. The folder “documentation” contains

The metadata dictionary (as in this Supplementary Material), in .txt and .json formats

The “dataset-description.json”, with the required description fields per BIDS specifications

The readme file, describing the general structure of the dataset

## Technical Validation

### Lesion delineation and agreement between lesion tracers

All the delineations were performed using ROIEditor (https://www.mristudio.org). A “seed growing” tool in ROIEditor was often used to achieve a broad segmentation, followed by manual adjustments. The segmentation was performed by two individuals highly experienced (more than 10 years) in lesion tracing (JH, XX). Additionally, they were trained by detailed instructions and illustrative files, in a subset of 220 cases (10% of the dataset cases with lesions). These cases were then revised by a neuroradiologist (AVF), discussed with the evaluators, and retrace and revised after 2 weeks. After achieving consensus, the evaluators started working on the whole dataset. The neuroradiologist revised all the segmentations and identified the suboptimum cases that were re-traced. The segmentations were revised as many times as necessary, until reaching final decision by the consensus of the tracers and the neuroradiologist. In the ischemic lesions, the evaluators looked for hyperintensities in DWI and/or hypointensities (<30% average brain intensity) in ADC. Additional contrasts were used to rule out chronic lesions or microvascular white matter disease. In the hemorrhage “lesion type”, extra modalities (SWI, T1WI, T2WI, FLAIR) were used to trace, in addition to DWI. Extra-parenchymal hemorrhage (intraventricular or subarachnoid) was not traced. The mean time for tracing was 7 min. The lesion definition was saved as a binary mask (lesion = 1, background = 0), in the original image space of each subject.

We calculated inter-and intra-rater reliability using the Dice similarity coefficient, which indicates if the same voxels are being selected as part of the lesion mask or not. Values range between 0 and 1 (1 is total agreement). For Dice calculation, we used the set of 220 lesions traced twice. This sample had the same proportion of ischemic and hemorrhagic lesions as the whole sample. The inter-rater Dice was 0.68 ± 0.23, while the intra-rater Dice was 0.72 ± 0.14. The agreement was better in ischemic lesions (0.76 ± 0.14 inter-evaluator and 0.79 ± 0.12 intra-evaluator) compared to hemorrhage. We also calculated the intraclass correlation coefficient (ICC) for the lesion volumes. The ICC ranges from 0–1; 1 is total agreement. The inter- and intra-rater ICC were 0.96 and 0.98, respectively. We reinforce that the final decision for the lesion segmentation in the whole dataset was made after many revisions and by consensus between the tracers and an expert neuroradiolist.

### Automated skull stripping - brainmask network

The deep-learning method used for skull stripping, in order to reduce the complexity and computational time of the process, is described in our previous paper^39^. Briefly, to generate the gold standards brain masks, the DWI and B0 images from the “not-visible” cases were resampled into 1 × 1 × 1 *mm*^3^ and skull striped by a level set algorithm (available with ROIEditor), with *W*_5 = _1.2 and 4, respectively (see explanation about choice of parameters in MRIstudio.org). The resulting brain masks (the union of masking on DWI and B0) were manually corrected by our annotators, serving as ground true for the “UNet BrainMask Network”. To train the network, all images are mapped to MNI and down-sampled to 4 × 4 × 4 *mm*^3^. The final brain mask inferenced by the network was then post-processed by the closing and the “binary_fill_holes” functions from Python scipy module, upsampled to 1 × 1 × 1 *mm*^3^, and dilated by one voxel with image smoothing. The Dice agreement between the “gold-standard” brain masks and those obtained with our network was above 99.9%, in an independent test set. The average processing time was about 19 seconds (against 4.3 min taken by the level-set algorithm), making it suitable for large scale, fast processing.

### DWI intensity normalization

The process described here is similar to that descried in our previous publication^39^, now extended to the whole dataset, including the cases with hemorrhage lesions.

Intensity-normalization increases the comparability between subjects and, as normalizing images to a standardized space, is crucial for diverse image analytical processes. Although the lesion might affect intensity distribution, we assume that the majority of brain voxels are from healthy tissue and can be a good reference for intra- and inter-individual comparison. We used bimodal Gaussian function, as in^42^, in Eq. (1) to fit the intensity histogram of DWI and cluster two groups of voxels: the “brain tissue” (the highest peak) and “non-brain tissue” (the lowest peak at lowest intensities, composed mostly by cerebrospinal fluid).

1 $$f(x)={a}_{1}{\exp }\left(-{\left(\frac{x-{b}_{1}}{{c}_{1}}\right)}^{2}\right)+{a}_{2}{\exp }\left(-{\left(\frac{x-{b}_{2}}{{c}_{2}}\right)}^{2}\right)$$

where *a*_*i*_*, b*_*i*_*, c*_*i*_ are the coefficients of the scale, mean, and standard deviation of Gaussian distribution. *a*_*i*_*, b*_*i*_*, c*_*i*_ are calculated by least-square fitting the bimodal Gaussian function to the intensity histogram of individual DWI. DWI intensities are normalized to make the “brain tissue” intensity with zero mean and one standard deviation.

**Fig. 5:**
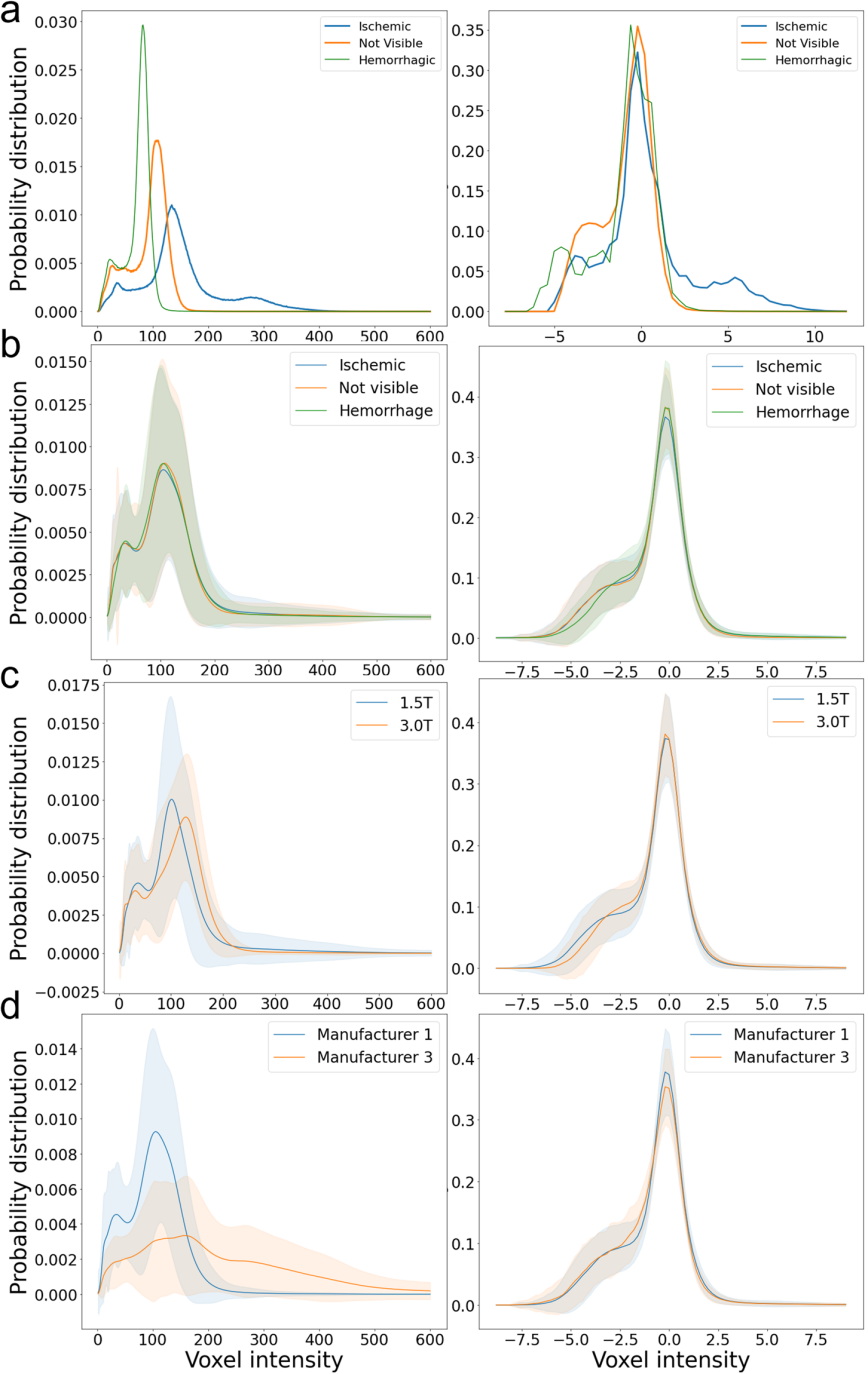
Probability distribution (y axis) of DWIs’ voxel intensity (x axis) prior-to (left column) and post-to (right column) intensity normalization. Panel (**a**) shows the distributions of DWI intensities of a selected cases with ischemic lesion (blue), hemorrhage (green), and “not visible” lesion (orange). Panels (**b**), (**c**), and (**d**) show the distributions of DWI intensities in groups according to presence of visible ischemic abnormality or hemorrhage (**b**), magnetic fields (**c**), and scanner manufacturers (**d**). The solid line is the average group distribution, the shadowed area is within 1 standard deviation from average.

Figure 5a show that the DWI intensity distribution of voxels in a brain with ischemic lesions (blue), one with hemorrhage (green), and in a brain with “not visible” lesion (orange), prior-to (left column) and post-to (right column) intensity normalization. We note that the preservation of the minor peak at high intensities in the brain with ischemic lesion indicates the preservation of the lesion contrast after normalization. Figure 5b–d show the distribution of DWI intensities in groups of images, prior-to (left column) and post-to (right column) intensity normalization. We note that the distributions are much more homogeneous, and the individual variations are smaller after intensity normalization. More importantly, intensity differences between different magnetic fields and scan manufacturers are ameliorated after intensity normalization.

We also note that this normalization approach helped to reduce the complexity and time to train Deep Learning Networks for ischemic lesion segmentation. We experimented training UNet with DWIs normalized by our proposed method (‘ProposedNorm’) and three others: (1) standard z-score normalization on whole images (‘StandardNorm’), (2) standard z-score normalization on brain-masked region only (‘BrainMaskStandardNorm’), and (3) Max-Min normalization (‘MaxMinNorm’). We kept all other procedures for training the network, inferencing predicts, and the post-processing the same, as described in^39^. We used the intensity normalized DWI and ADC as inputs, and 5-fold cross-validation. Table 2 shows that the Dice scores between automated and manually traced images were higher when using images intensity-normalized with the proposed method.

Finally, in addition to the ADC from the scanners, we offer ADCs “recalculated” as:

2 $${I}_{ADC}(x,y,z)=\frac{lnI(x,y,z)-ln{I}_{0}(x,y,z)}{b}$$

where *I*(*x, y, z*), *I*_0_(*x, y, z*) are the intensity of DWI and B0 voxels, respectively, at (*x, y, z*)-coordinate, with b-value = 1000.

### Quality control for images in standardized space, MNI

Clinical images offer extra challenges for brain mapping to standardized space, because of their high slice thickness and fair amount of tilt out-of-plane, therefore requiring further stringent quality control. The quality control of the normalized images was performed in two steps: (1) qualitative: a neuroradiologist looked at the MNI-normalized images with the MNI template brain mask overlaid, in order to rule-out obvious misalignments, and (2) quantitative: performed as described below.

**Fig. 6:**
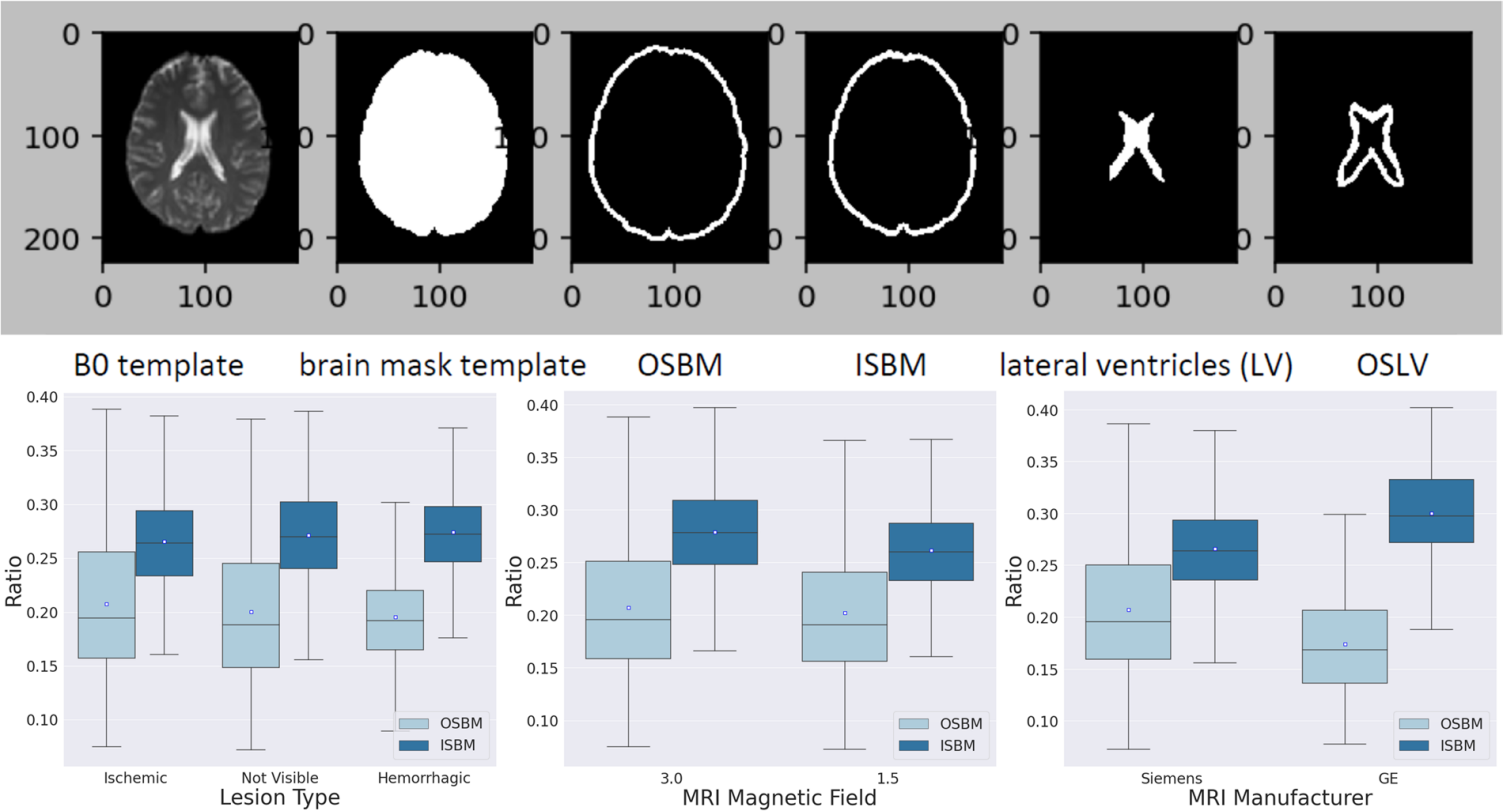
Quality control of the image mapping to standard coordinates (MNI). The top figure illustrates the three regions of 5 voxels bandwidth defined in the template: the outside strip of the brain mask (OSBM), the inside strip of the brain mask (ISBM), and the outside strip of the lateral ventricles (OSLV). The bottom boxplots illustrate the average of OSBM and ISBM (γ_OSBM_ and γ_ISBM_), which are indicative of the global quality of the brain mapping, across lesion type, magnetic field and scanner manufacturer.
- The ratio of the mis-deformed voxels in OSBM, ISBM and OSLV for each subject, defined as γ_OSBM_, γ_ISBM_, and γ_OSLV_, was calculated as follows:

- Three regions of 5 voxels bandwidth were defined in the template: the outside strip of the brain mask (OSBM), the inside strip of the brain mask (ISBM), and the outside strip of the lateral ventricles (OSLV), as shown in Fig. 6, top.

3 $${\gamma }_{{\rm{OSBM}}}=\frac{{\rm{the}}\;{\rm{number}}\;{\rm{of}}\;{\rm{the}}\;{\rm{deformed}}\;{\rm{B0}}\;{\rm{voxels}}\;{\rm{whose}}\;{\rm{intensity}}\;{\rm{is}}\;{\rm{larger}}\;{\rm{than}}\;{\rm{0}}\;{\rm{in}}\;{\rm{OSBM}}}{{\rm{the}}\;{\rm{number}}\;{\rm{of}}\;{\rm{voxels}}\;{\rm{in}}\;{\rm{OSBM}}}$$

4 $${\gamma }_{{\rm{ISBM}}}=\frac{{\rm{the}}\;{\rm{number}}\;{\rm{of}}\;{\rm{the}}\;{\rm{deformed}}\;{\rm{B0}}\;{\rm{voxels}}\;{\rm{whose}}\;{\rm{intensity}}\;{\rm{is}}\;{\rm{0}}\;{\rm{in}}\;{\rm{ISBM}}}{{\rm{the}}\;{\rm{number}}\;{\rm{of}}\;{\rm{voxels}}\;{\rm{in}}\;{\rm{ISBM}}}$$

5 $${\gamma }_{{\rm{OSLV}}}=\frac{{\rm{the}}\;{\rm{number}}\;{\rm{of}}\;{\rm{the}}\;{\rm{deformed}}\;{\rm{B0}}\;{\rm{voxels}}\;{\rm{whose}}\;{\rm{intensity}}\;{\rm{is}}\;{\rm{larger}}\;{\rm{than}}\;\lambda \;{\rm{in}}\;{\rm{OSLV}}}{{\rm{the}}\;{\rm{number}}\;{\rm{of}}\;{\rm{voxels}}\;{\rm{in}}\;{\rm{OSLV}}}$$

6 $$\lambda ={\mu }_{{\rm{deformed}}{\rm{B0}}{\rm{in}}{\rm{LV}}}-0.5\times {\sigma }_{{\rm{deformed}}{\rm{B0}}{\rm{in}}{\rm{LV}}}$$

γ_OSBM_ indicates the ratio of the deformed B0 voxels aligned outside the template brain mask and γ_ISBM_ indicates the ratio of the background voxels aligned inside the template brain mask. High γ_OSBM_ or γ_ISBM_ indicte possible issues with the global brain mapping. γ_OSLV_ indicates the ratio of voxels from a subject’s deformed ventricles that exist outside the template lateral ventricle boundaries. High γ_OSLV_ is common in this population since aged subjects’ lateral ventricles are usually larger than the template’s lateral ventricles and indicate the need for local, and possibly non-linear deformation for brain mapping.

The average γ_OSBM_ was 0.2042 ± 0.0628 (medium = 0.1929, range [0.0723, 0.5070]). The average γ_ISBM_ was 0.2683 ± 0.0426 (medium = 0.2666, range [0.1560, 0.4273]). This means that a small minority of voxels were outer or inner the template brain contour, when considering a stringent bandwidth of 5 voxels. Importantly, the small ratio of error was stable over stroke types, magnetic field, or scan manufacturer (Fig. 6).

## Usage Notes

Multimodal public repositories for research data have been organized on the pillars of “FAIR” principles^28^: they are designed to be maximally “Findable, Accessible, Interoperable, and Reusable”. These repositories and centralized collections^43–47^, combined with initiatives to establish semantic and analytical consensus^48,49^, are likely to represent the core structure for future neuroscience research. The sharing of clinical data, however, is complicated by technical and regulatory issues. While the models of research data sharing are not adoptable in their exact same form for clinical data, they inspire a similar sharing structure, respecting the conditions under which the data are usable, without limiting accessibility.

We share a large dataset of clinical acute stroke MRIs, associated to demographic and clinical metadata, in alignment with the broad aim of the biomedical community to share FAIR data. Although a challenge for imaging processing, the image heterogeneity is an important feature of the dataset as it guarantees that tools developed using these images can be applied broadly. Providing multimodal image data is another important achievement as it will enable to train and test models that rely in multimodal integration and/or data fusion. The data organization, in BIDS^36^ recommended format, is compatible, or can be easily converted to, newly developed semantic standards, such as the NeuroImaging Data Model (NIDM)^48^. This provides critical capability to generate “computable data objects”, that can be readily used by the AI community and are user-friendly organized to improve access to non-expert data analysts. It also makes easy to integrate with other ongoing open science efforts^29,43^, analytical pipelines (such as in brainlife, https://brainlife.io/about/), application program interfaces (APIs), modules for quality control^50,51^ and harmonization^52–54^, and indexing and management engines^55^. These capabilities enable the use of this resource not only for discovery, but also for data synthesis and augmentation^56–58^, and to aid reproducibility and replication studies^59,60^.

Specifically, the dataset presented here could be used to train, test, or “transfer learning” to algorithms for lesion segmentation, providing highly important metrics for acute treatment, such as the volume of the ischemic core and perfusion deficits. For example, we recently used the images with ischemic strokes from this dataset to developed a public, user-friendly tool to generate “computable data objects”^61^ (https://www.nitrc.org/projects/ads). We also developed a public, user-friendly tool for ischemic lesion segmentation and quantification^39^, overcoming limitations of previous algorithms not tested in large numbers of real clinical data^62,63^. We created the first public digital atlas of brain arterial territories^64^ (https://www.nitrc.org/projects/arterialatlas), based on the frequency lesion maps of 1,298 of these cases. This dataset could also be used to develop and test algorithms for mapping low resolution images of brains with lesions. This mapping allows the examination of the overlap of the lesion with specific brain structures, like those defined in our arterial territory atlas or others. This enables voxel-based lesion symptom mapping and the automated calculation of relevant scores, as for example, we did using this dataset by automatically estimating ASPECTS^65^. The association of image annotations and lesion description also enabled us to develop automated image retrieval engines and to generate automated radiological reports^66^. Furthermore, this dataset is potentially useful as a general training and testing resource for translational research. In fact, we initiate several of those efforts by using this dataset and the tools enabled through it to study laboratory^67^ and anatomic-functional relations^68^, to explore bias in clinical measures^69^, to study populational trends^70^, and to test hypothesis developed in external, smaller datasets.

The main limitation of this dataset is that it originates from a single center. Although we used data from a certified Comprehensive Stroke Center, whose population reflects the profile of the US national population with stroke, and scans with great technical heterogeneity (collected along 10 years, in eleven scanners, and with dozens of different protocols), a regional bias^71^ might exist. For instance, our population includes a higher percentage of Black and lower percentage of Hispanic/Latinx and Asian patients than many urban stroke centers. We expect that future sharing and indexing initiatives enable the enrichment of this dataset with data from multiple other centers, worldwide, reducing possible population biases. Nevertheless, this dataset will serve as a valuable resource for training and testing models, particularly those for technical development and for image processing.

### Supplementary information

Supplementary Information

## Data Availability

No custom code was used.
