# Supplementary material for: A large public dataset of annotated clinical MRIs and metadata of patients with acute stroke
Source: Sci Data. 2023 Aug 22;10:548. doi: 10.1038/s41597-023-02457-9 (PMC10444746; doi:10.1038/s41597-023-02457-9)
Supplement: Supplementary file 1 — Supplementary Information [file 41597_2023_2457_MOESM1_ESM.docx]

**Supplementary Material: Metadata Dictionary**

Age

Sex

Race

Diagnosis

Description age of the participant

Units years (<30 for 30 years-old and younger, >90 for 90 years-old and older)

Description sex of the participant reported by the participant Levels M: male

F: female

Description race of the participant reported by the participant Levels BAA: Black or African American

W: white A: Asian O: other

NA: not available

Description clinical diagnosis at admission Levels IS: Ischemic Stroke

ES: Embolic Stroke

ICH: Intracranial Hemorrhage TIA: Transitory Ischemic Accident SAH: Subarachnoid Hemorrhage

Medical-history

Description previous medical conditions reported by the participant or available on medical charts at admission

Levels Hypertension: history of systolic blood pressure >=130mmHg or diastolic

>=80mmHg or use of anti-hypertensive medication

Dyslipidemia: medical history of dyslipidemia or use of anti-cholesterol medication Diabetes Mellitus: history of diabetes or use of anti-glucose medication

Smoker: smoke status as reported by the participant

Previous Stroke: history of stroke as reported by the participant or by medical chart CAD/prior MI: Coronary arterial disease or prior myocardial infarct

Previous TIA: history of transitory ischemic accident

Family History of Stroke: first relative history of stroke as reported by the participant Atrial Fib/Flutter: history of Atrial Fibrillation or atrial Flutter

HF: Heart Failure as reported by the participant or by medical chart Prosthetic Heart Valve: history of Prosthetic Heart Valve

Renal Insufficiency – chronic: renal insufficiency as reported by participant or by chart

Migraine: history of migraine as reported by participant

Obesity / Overweight: obesity or overweight reveled by history of BMI at admission PVD: history of peripheral vascular disease

Prior-medication

Carotid Stenosis: history of carotid stenosis

Sleep Apnea: history of sleep apnea as reported by the participant or by medical chart

Description previous use of listed medications as reported by the participant or available on medical charts at admission

Levels Antihypertensive: Antihypertensive drug Antiglucose: Antiglucose drug Anticholesterol: Anticholesterol drug Antiplatet: Antiplatet drug Anticoagulant: Anticoagulant drug

Ambulation-prior

Description ability to walk prior to the admission as reported by the participant

Levels 1: Able to ambulate independently (no help from another person, w/ or w/o device) 2: With assistance (from person)

NIHSS

3: Unable to ambulate

Description The National Institutes of Health Stroke Scale at admission exam TermURL h[ttps://www.](http://www.stroke.nih.gov/documents/NIH_Stroke_Scale_508C.pdf)strok[e.nih.gov/documents/NIH_Stroke_Scale_508C.pdf](http://www.stroke.nih.gov/documents/NIH_Stroke_Scale_508C.pdf)

Ambulation-arrival

Description ability to walk at hospital arrival

Levels 1. Able to ambulate independently (no help from another person, w/ or w/o device)

1. With assistance (from person)
2. Unable to ambulate

IVtPA

Systolic

Diastolic

Description Thrombolysis with intravenous tissue-type plasminogen activator performed before the MRI scan in the dataset

Levels 1. Yes

1. No

Description systolic blood pressure at hospital arrival Units mm Hg

Description systolic blood pressure at hospital arrival Units mm Hg

Cholesterol

Description total blood cholesterol level at first laboratorial exam after hospital arrival Units mg/dL

Triglycerides

Description triglycerides level at first laboratorial exam after hospital arrival

HDL

LDL

hba1c

Glucose

Creatinine

Units mg/dL

Description High-density lipoprotein cholesterol level at first laboratorial exam Units mg/dL

Description Low-density lipoprotein cholesterol level at first laboratorial exam Units mg/dL

Description Hemoglobin A1c at first laboratorial exam after hospital arrival Units %

Description Blood fasting glucose level at first laboratorial exam after hospital arrival Units mg/dL

Description Serum creatinine level at first laboratorial exam after hospital arrival Units mg/dL

Prothrombin

Description Prothrombin time at first test after hospital arrival Units International Normalized Ratio, INR

BMI

Description Body mass index of the participant Units kg/m2

Ambulation-discharge

Description ability to walk at hospital discharge

Levels 1. Able to ambulate independently (no help from another person) w/ or w/o device)

- 1. With assistance (from person)
  2. Unable to ambulate

Hospitalization days

Description Time of hospital stay Units days

Symptoms-MRI

Description Hours between symptoms and MRI scan. Only available for patients with ischemic strokes highly confident about the symptom’s onset

90days-mRS

Units Hours (>72 for more than 72h between scan and symptoms onset)

Description The modified Rankin Scale 90 days after the acute stroke Levels 0. No symptoms

1. No significant disability. Able to carry out all usual activities, despite some symptoms
2. Slight disability. Able to look after own affairs without assistance, but unable to carry out all previous activities
3. Moderate disability. Requires some help, but able to walk unassisted
4. Moderately severe disability. Unable to attend to own bodily needs without assistance, and unable to walk unassisted

Lesion-type

1. Severe disability. Requires nursing care and attention, bedridden, incontinent
2. Dead

Description Stroke lesion appearance at MRI, according to radiological diagnosis

Levels ischemic: DWI hyperintensity; ADC hypo / isointensity, per radiological interpretation hemorrhagic: any signal of bleeding, intra- or extra-parenchymal, per radiological interpretation. It includes primarily hemorrhagic strokes and hemorrhagic transfor- mation of ischemic strokes.

not visible: ischemic area is not visible, per radiological interpretation. It does not include retrospective detection (after a follow up scan or automated detection).

Side

Description Stroke hemisphere according to MRI

MR-field

Levels Left Right Bilateral

Description Magnetic Field of the MR scan, in tesla Levels 3

MR-manufacturer

1.5

Description MR scan manufacturer Levels 1. Siemens

Other-MRI-modalities

1. Phillips
2. GE
3. Not Recorded

Description Available MRI modalities, in addition to the DWI Levels ADC: apparent diffusion coefficient

FLAIR: Fluid-attenuated inversion recovery, defaced if high resolution out-plane MPRAGE: high resolution T1weighted image, defaced

PWI: perfusion weighted image SWI: susceptibility weighted image

T1w: T1-weighted images, defaced if high resolution out-plane T1wC: T1-weighted images, post-contrast iv injection

T2w: T2-weighted images, defaced if high resolution out-plane

Lesion-volume

Description Volume of the stroke (number of voxels x voxel size) as described by the manual delineation of the MRI lesion

Units mm3

Supplementary Table 1: Summary of image parameters (values presented as median (interquartile range, IQR))

|  | **DWI** | **PWI** | **FLAIR** | **MPRAGE** | **T1w** | **T2w** | **SWI** |
| --- | --- | --- | --- | --- | --- | --- | --- |
| In Plane voxel size (mm) | 1.20 (0.90, 1.20) | 1.80 (1.72, 1.88) | 0.72 (0.72, 0.86) | 1.00 (0.90, 1.50) | 0.86 (0.69, 0.90) | 0.86 (0.69, 0.90) | 0.898 (0.859, 0.898) |
| Slice Thickness  (mm) | 5.00 (4.00, 5.00) | 5.00 (4.00, 5.00) | 4.00 (4.00, 4.75) | 1.00 (0.90, 1.50) | 4.00 (4.00, 5.00) | 4.00 (4.00, 5.00) | 2.00 (1.50, 2.00) |
| Slice Thick. w/  Gap (mm) | 5.00 (4.00, 6.00) | 6.50 (5.20, 7.00) | 4.00 (4.00, 5.00) | 0.98 (0.90, 0.98) | 5.00 (4.00, 5.00) | 5.00 (4.00, 5.00) | 2.00 (1.50, 2.00) |
| Number of  Slices | 32 (25, 40) | 20 (18, 22) | 40 (32, 40) | 256 (256, 256) | 35 (30, 40) | 35 (30, 40) | 80 (72, 96) |
| Field of View x  (mm) | 192 (192, 256) | 128 (128, 128) | 250 (240, 256) | 176 (104, 192) | 256 (256, 320) | 256 (256, 320) | 224 (208, 232) |
| Field of View y  (mm) | 192 (192, 256) | 128 (128, 128) | 320 (256, 320) | 256 (256, 256) | 256 (256, 320) | 256 (256, 320) | 256 (256, 256) |
| Acquisition  Matrix | 192 (160, 192) | 128 (128, 128) | 184 (180, 197) | 246 (246, 256) | 205 (189, 256) | 205 (189, 256) | 177 (177, 205) |
| Reconstruction  Matrix | 192 (192, 256) | 128 (128, 128) | 250 (240, 270) | 256 (256, 496) | 256 (256, 320) | 256 (256, 320) | 224 (208, 232) |
| Repetition  Time (ms) | 7.50 (6.50, 9.00) | 1.61 (1.41, 1.67) | 9.00 (9.00, 9.00) | 1.90 (1.90, 2.11) | 0.55 (0.40, 0.61) | 0.55 (0.40, 0.61) | 0.048 (0.027, 0.049) |
| Echo Time  (ms) | 0.094 (0.087,  0.098) | 0.030 (0.030,  0.040) | 0.105 (0.100,  0.114) | 0.0027 (0.0027,  0.0033) | 0.009 (0.005,  0.010) | 0.009 (0.005,  0.010) | 0.040 (0.020, 0.040) |
| Flip Angle  (degree) | 90 (90, 90) | 90 (90, 90) | 154 (150, 160) | 15 (8, 15) | 90 (90, 130) | 90 (90, 130) | 15 (15, 15) |
| Inversion Time  (ms) | NA | NA | 2.50 (2.50, 2.50) | 1.10 (0.90, 1.10) | NA | NA | NA |
| Number of  Frames | NA | 60 (50, 60) | NA | NA | NA | NA | NA |


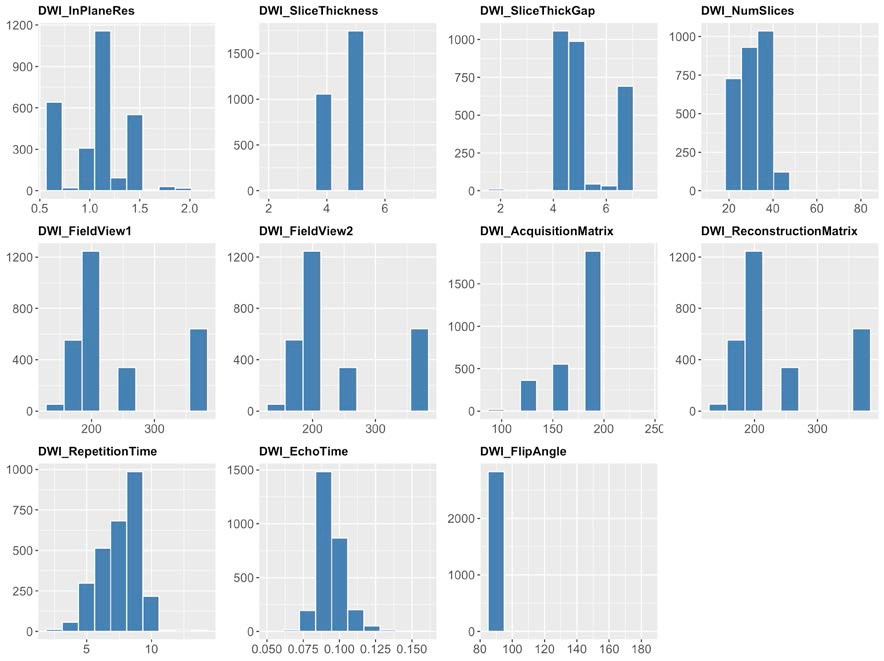


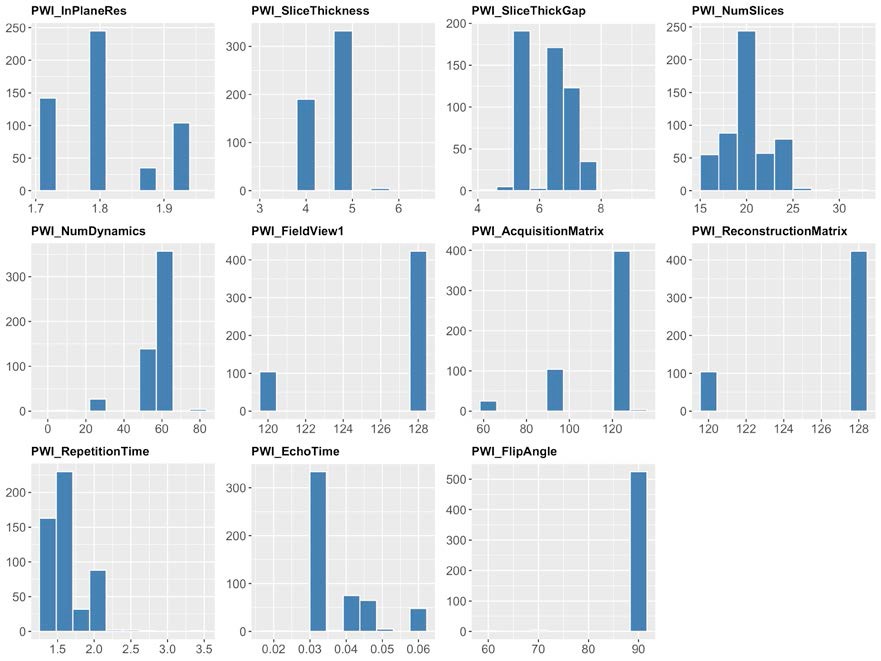


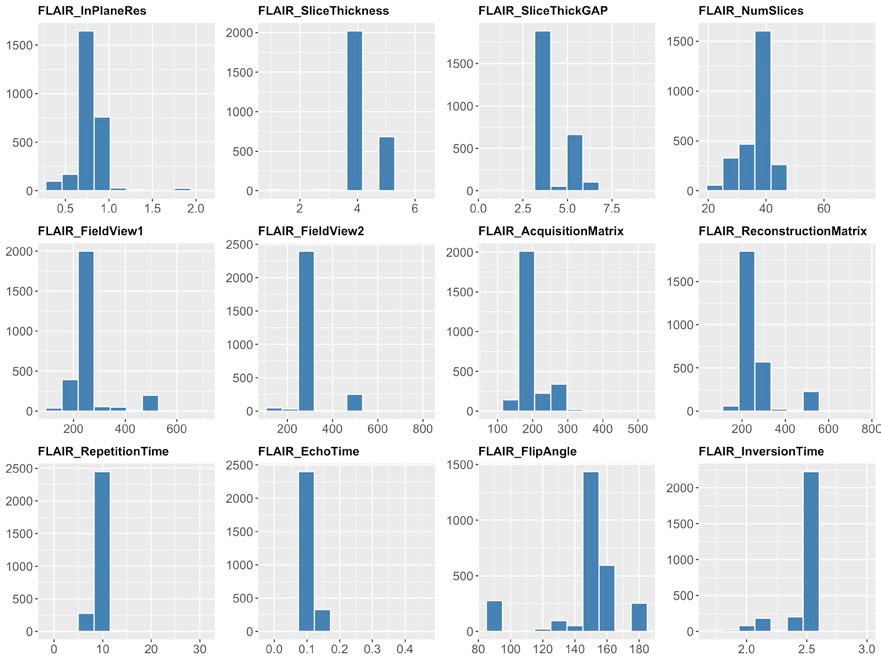


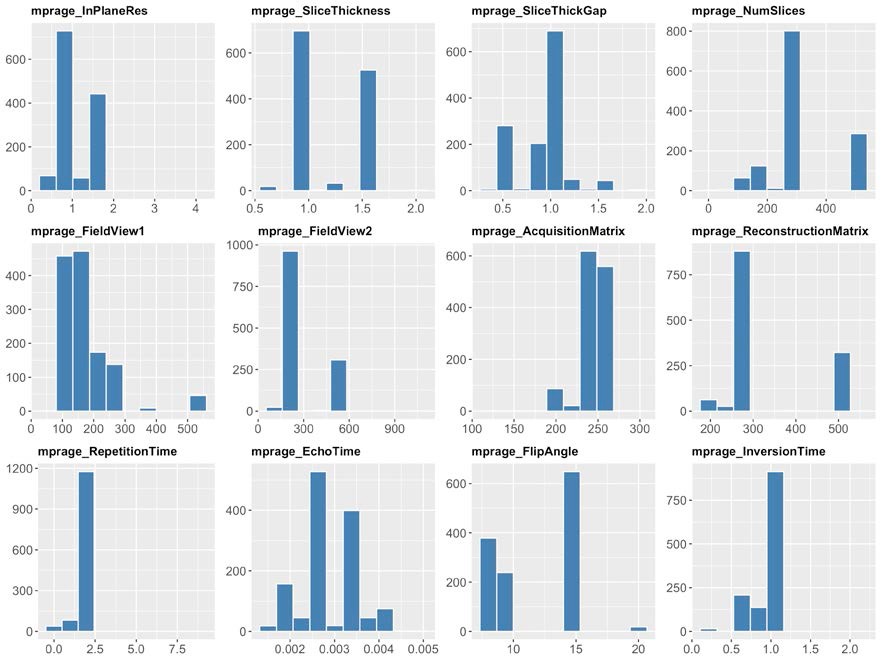


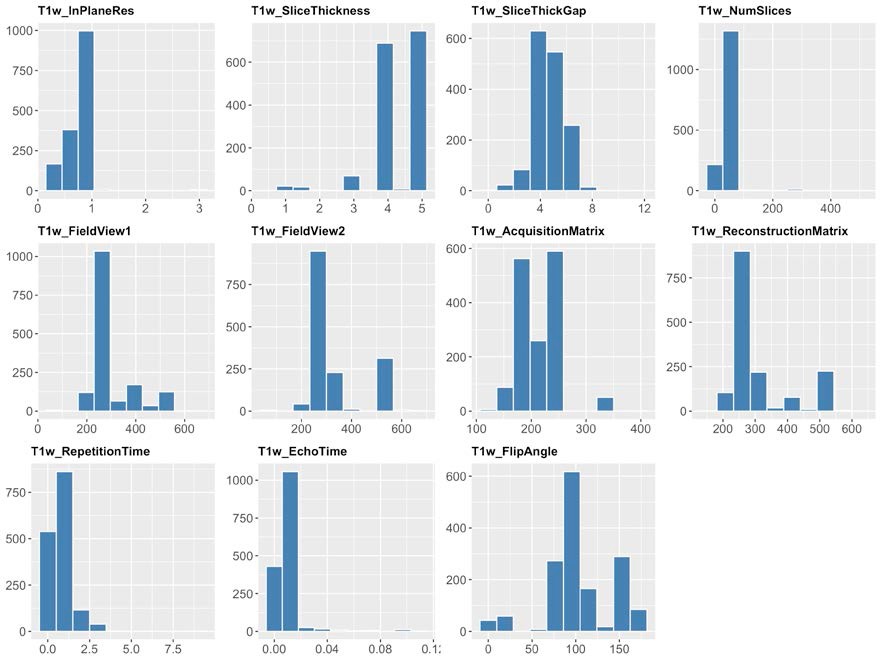


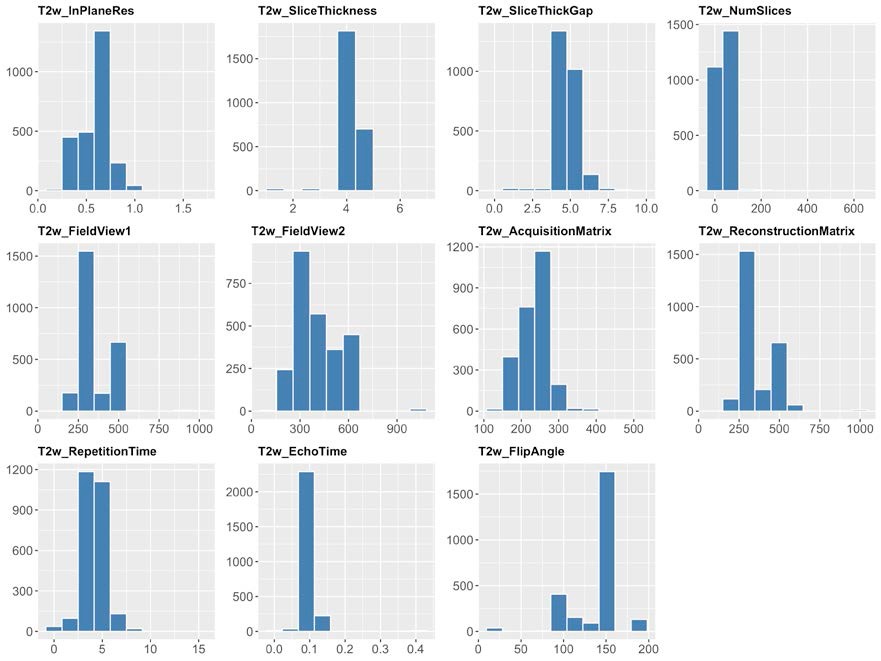


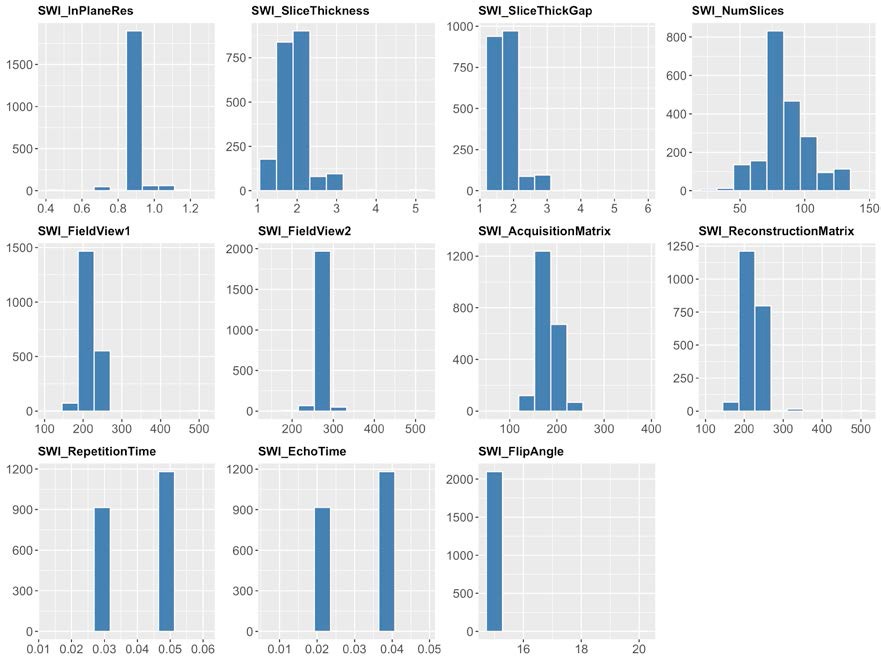


Supplementary Figure 1: Histograms of image parameters. InPlaneRes is the in plane voxel size in mm. Slice Thickness and Field of View are in mm. Echo Time, Repetition Time and Inversion Time are in ms.
